# Supplementary material for: A fault-intrusion-tolerant system and deadline-aware algorithm for scheduling scientific workflow in the cloud
Source: PeerJ Comput Sci. 2021 Nov 2;7:e747. doi: 10.7717/peerj-cs.747 (PMC8576554; doi:10.7717/peerj-cs.747)
Supplement: Supplemental Information 6 [file peerj-cs-07-747-s006.zip › Myproject1.1/docs/org/workflowsim/scheduler/MinMinScheduler.html]

MinMinScheduler


JavaScript is disabled on your browser.


- Overview
- Package
- Class
- Use
- Tree
- Deprecated
- Index
- Help

- Prev Class
- Next Class

- Frames
- No Frames

- All Classes

- Summary:
- Nested |
- Field |
- Constr |
- Method

- Detail:
- Field |
- Constr |
- Method


org.workflowsim.scheduler

## Class MinMinScheduler

- java.lang.Object
- - org.workflowsim.scheduler.BaseScheduler
  - - org.workflowsim.scheduler.MinMinScheduler

- All Implemented Interfaces:
  :   SchedulerInterface

  ---

    

  ```
  public class MinMinScheduler
  extends BaseScheduler
  ```

  MinMin algorithm.

  Since:
  :   WorkflowSim Toolkit 1.0

- - ### Constructor Summary

    Constructors

    Constructor and Description

    `MinMinScheduler()`
  - ### Method Summary

    Methods

    | Modifier and Type | Method and Description |
    | --- | --- |
    | `void` | `run()` The main function |

    - ### Methods inherited from class org.workflowsim.scheduler.BaseScheduler

      `getCloudletList, getScheduledList, getVmList, setCloudletList, setVmList`
    - ### Methods inherited from class java.lang.Object

      `clone, equals, finalize, getClass, hashCode, notify, notifyAll, toString, wait, wait, wait`

- - ### Constructor Detail


    - #### MinMinScheduler

      ```
      public MinMinScheduler()
      ```
  - ### Method Detail


    - #### run

      ```
      public void run()
      ```

      **Description copied from class: `BaseScheduler`**

      The main function

      **Specified by:**
      :   `run` in interface `SchedulerInterface`

      **Specified by:**
      :   `run` in class `BaseScheduler`


- Overview
- Package
- Class
- Use
- Tree
- Deprecated
- Index
- Help

- Prev Class
- Next Class

- Frames
- No Frames

- All Classes

- Summary:
- Nested |
- Field |
- Constr |
- Method

- Detail:
- Field |
- Constr |
- Method
